# *Resilin* is needed for wing posture in *Drosophila suzukii*

**DOI:** 10.1002/arch.21913

**Published:** 2022-05-23

**Authors:** Steven Lerch, Yang Yang, Justin Flaven‐Pouchon, Nicole Gehring, Bernard Moussian

**Affiliations:** ^1^ Interfaculty Institute for Cell Biology Animal Genetics, Universität Tübingen Tübingen Germany; ^2^ Senckenberg Natural History Collections Dresden Germany; ^3^ INRAE, CNRS, Institut Sophia Agrobiotech, Université Côte d'Azur Nice France

**Keywords:** dityrosine, elastic cuticle, flight, resilin, wing

## Abstract

Resilin is a protein matrix in movable regions of the cuticle conferring resistance to fatigue. The main component of Resilin is Pro‐Rresilin that polymerises via covalent di‐ and tri‐tyrosine bounds (DT). Loss of Pro‐Resilin is nonlethal and causes a held‐down wing phenotype (*hdw*) in the fruit fly *Drosophila melanogaster*. To test whether this mild phenotype is recurrent in other insect species, we analysed resilin in the spotted‐wing fruit fly *Drosophila suzukii*. As quantified by DT autofluorescence by microscopy, DT intensities in the trochanter and the wing hinge are higher in *D. suzukii* than in *D. melanogaster*, while in the proboscis the DT signal is stronger in *D. melanogaster* compared to *D. suzukii*. To study the function of Pro‐Resilin in *D. suzukii*, we generated a mutation in the *proresilin* gene applying the Crispr/Cas9 technique. *D. suzukii pro‐resilin* mutant flies are flight‐less and show a *hdw* phenotype resembling respective *D. melanogaster* mutants. DT signal intensity at the wing hinge is reduced but not eliminated in *D. suzukii hdw* flies. Either residual Pro‐Resilin accounts for the remaining DT signal or, as proposed for the *hdw* phenotype in *D. melanogaster*, other DT forming proteins might be present in Resilin matrices. Interestingly, DT signal intensity reduction rates in *D. suzukii* and *D. melanogaster* are somehow different. Taken together, in general, the function of Pro‐Resilin seems to be conserved in the Drosophila genus; small differences in DT quantity, however, allow us to hypothesise that Resilin matrices might be modulated during evolution probably to accommodate the species‐specific lifestyle.

## Highlights

- Resilin/di‐tyrosine distribution in *Drosophila suzukii* and *Drosophila melanogaster* adults is similar.
- Reduction or elimination of Resilin/di‐tyrosine causes an identical phenotype in *D. suzukii* and *D. melanogaster*.
- Besides proresilin, resilin matrices in Drosophila species probably contain also other proteins.
- A general positive correlation between body size and Resilin/di‐tyrosine amounts is not apparent in Drosophila species.

## Graphical abstract

Di‐tyrosine represents proresilin in several patches in the cuticle of the wing hinge region in *Drosophila suzukii*. In proresilin mutant *D. suzukii* flies the di‐tyrosine signal is reduced indicating reduction or elimination of proresilin. Reduction or elimination of dityrosine and proresilin in *D. suzukii* causes wing posture defects preventing flight.

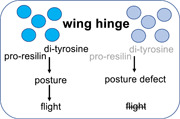

## 1 INTRODUCTION

The body cuticle of insects is a protein–chitin matrix (Moussian, 2010, 2013) and consists of hard and soft regions that together ensure movability of the organism. Usually, the degree of crosslinking to catecholamines (sclerotization) and the type of chitin‐binding proteins define the hardness of the cuticle. Besides, some regions of the cuticle need to be adhesive, elastic, extendible or resistant to fatigue, properties that are considered to be mainly conferred by the resilient protein Pro‐Resilin that polymerises through covalent di‐ and tri‐tyrosine bounds (DT). Resilin matrices were discovered in 1960 by F. Weis‐Fogh in the prealar arm and the wing hinge of the locust *Schistocerca grigaria* and the elastic tendon of the dragonfly *Aeshna grandi* (Weis‐Fogh, 1960). Subsequently, Resilin matrices were identified in a number of species (Michels & Gorb, 2012). The main method for Resilin identification has become its, albeit indirect, detection by fluorescence microscopy, through which the autofluorescence of DTs after excitation with UV light is visualised and quantified. Using a GFP‐tagged version of Pro‐Resilin in *Drosophila melanogaster*, Pro‐Resilin is found in the spermathecal ducts, the basis of bristles, the proboscis, the tracheal endings, close to the leg joints and the wing hinges (Lerch et al., 2020). Most of these signals overlap with the DT signal. Interestingly, mutations in the *D. melanogaster proresilin* gene are not lethal but cause a wing posture failure compromising flight and courtship. This calls for proteins in the Resilin matrix that may compensate for Pro‐Resilin elimination of reduction. Indeed, in various insect species, more than one Pro‐Resilin coding genes have been annotated. In the bed bug *Cimex lectularius*, for instance, at least six orthologues of Pro‐Resilin have been identified (Rosenfeld et al., 2016). These findings underline that Resilin matrices do not consist of a single protein. The quality of Resilin matrices, hence, might differ between different regions of the body and between species.

A central question in the field is, thus, the evolution of Resilin matrices within and between species. To test to what extent our findings in *D. melanogaster* are transferable to other insects, as the next step in our Resilin research, we decided to study the effects of mutations in the *proresilin* gene of the distantly related *Drosophila suzukii* (about 5 million years apart from *D. melanogaster*, Suvorov et al., 2022). Here, we report on the phenotype caused by such a mutation generated by the gene editing Crispr/Cas9 (CC) method.

## 2 MATERIALS AND METHODS

### 2.1 Fly husbandry

Wild‐type *D. suzukii* flies were captured in Tübingen, Germany, on blackberries in 2018. In the laboratory, they were reared on standard *Drosophila* food containing agar, corn grist, soja meal, dried yeast, beat syrup, malt extract, propionic acid and Nipagin at 22°C. Fresh baker's yeast was smeared on the hard food and a filter paper was stuck into the food to allow flies resting and to avoid them sticking to the food when larvae softened it.

### 2.2 CRIPSR/Cas9 experiments

To mutagenize the *proresilin* gene (DS10_00003274 in the database spottedwingflybase.org) in *D. suzukii*, we injected a Cas9 protein (150 ng/μl, NEB) together with the gDNA #4 (GACGCTGCTCATGGCAATGGTGG; 400 ng/μl) into preblastoderm embryos (Lerch et al., 2020). The gDNA sequence is 100% conserved between *D. suzukii* and *D. melanogaster*. We had used it in our previous work for successful mutagenesis of *proresilin* in *D. melanogaster*. According to the *flycrispr* software (https://flycrispr.org) prediction, no off‐target sites are present in the genome. After mass crossing of the flies generated by injected eggs, we continuously checked for the occurrence of the held‐down wing phenotype in our *proresilin^CC^*  stock. Flies with this phenotype were isolated and kept together to generate a stock for further analyses. For determination of the mutation, genomic DNA was prepared from these and control flies by standard methods to amplify and sequence the respective region using the primers ATTCCGATCAGCAGCAGTCC and GTCCGCCATTACCATTACCG. Sequences, determined by Microgen Europe B.V., were analysed by the BLAST software at the NCBI and the SignalP 6.0 software on the DTU Health Tech site.

### 2.3 Microscopy

For body size measurements, flies were submerged in halocarbon oil (Sigma‐Aldrich) on a glass slide and photographed on a Nikon AZ100 microscope at a magnification of 10x using the in‐built NIS software that allows length determination.

For morphological analyses, flies were observed on an inverse Axio Observer Z1 (Zeiss) equipped with an Axiocam Mono camera at the systems biology group of C. Dahmann at the TU Dresden. The filters were: AF405 (DT specific filter): Ex: 357/44 nm Brightline HC, splitter HC BS 389 nm, Em: 420/40 nm ET Bandpass; DAPI: Ex: 359/48 nm, splitter: 395 nm, Em: 445/50 nm; and GFP: Ex: 470/40 nm, splitter: 495 nm, Em: 525/50 nm. These objectives were used: EC Plan‐Neofluar 5x/0.67 M27 DRY, EC Plan‐Neofluar 10x/0.3 M27 DRY, and EC Plan‐Neofluar 20x/0.5 M27 DRY. The light source was a halogen lamp. Flies were immobilised by freezing at −20°C for 4 min, then dissected and mounted in ROTI®mount Aqua. Images were taken with the Zeiss inbuilt software. They were prepared using ImageJ and Adobe Illustrator CS6.

In this study, we aimed at identifying Resilin matrices indirectly by DT autofluorescence. For this purpose, we used a specific filter system adjusted to the published emission range (Anderson, 1964; Elvin et al., 2005; Michels & Gorb, 2012, see below). To not only measure the intensity detected by the specific DT‐filter, we relied (a) on our experiences with the co‐distribution of ResilinGFP and DT (Lerch et al., 2020), and (b), especially for the wing hinge, the differences between the intensities obtained with the specific DT filter and the DAPI filter (that covers a broader range including background) using the profile‐function of the ZenBlue (Zeiss internal) software. This approach allowed a more confident and accurate determination of the measured areas. In practice, we used the trochanter signal for calibration by extraction of the background (DAPI) signal from the DT signal. Absence of DT signal was assured by detection of the background in body areas without Rresilin (data not shown). For the counting of the wing hinge dots per individual, we set up a threshold of the mean value of the *D. melanogaster* wild‐type wing hinge signal extracted by the profile‐program comparing the intensities obtained by the DT and DAPI filters (DT > DAPI). The threshold of 7500 counts was used to produce the numbers of DT dots in the wing hinges of all used lines. These area counts where than compared and calculated with the total individual numbers (*n*) per line.

### 2.4 Statistics and determination of the correlation coefficient

Data were analysed using Student's *t*‐tests or ANOVAs followed by a Tukey HSD post hoc procedures. Normality and homoscedasticity of the samples were preliminarily assessed using Shapiro and Leven test, respectively. Data are represented using box‐blot indicating minimum, first quartile, median, third quartile, maximum, and the mean (red crosses). All statistical analyses were performed using XLstat 2020 (AddinSoft®). All computed *p*‐values are listed in Supporting Information: Data S4.

To estimate the correlation between body size and DT intensity, we have conceived a simple calculation method: subtractive correlation coefficient “body size: DT intensity” C^δs:dt^ = (mean body size species 1/mean intensity species 1) – (mean body size species 2/mean intensity species 2). If there is a body size‐proportional correlation (bigger body with proportionally stronger DT intensity), the coefficient approximates 0. If the coefficient is different from 0, we suppose a nonbody size dependent difference of intensity. Indeed, for most cases tested in this study, we obtain a coefficient of approximately 0 (Table 2 and Supporting Information: Data S4). As different populations of flies were analyzed in the body size measurements and the DT intensity determination experiments, a statistical testing of the C^δs:dt^ is not possible in this study.

## 3 RESULTS AND DISCUSSION

### 3.1 Resilin distribution in *D. suzukii*

**Figure 1:**
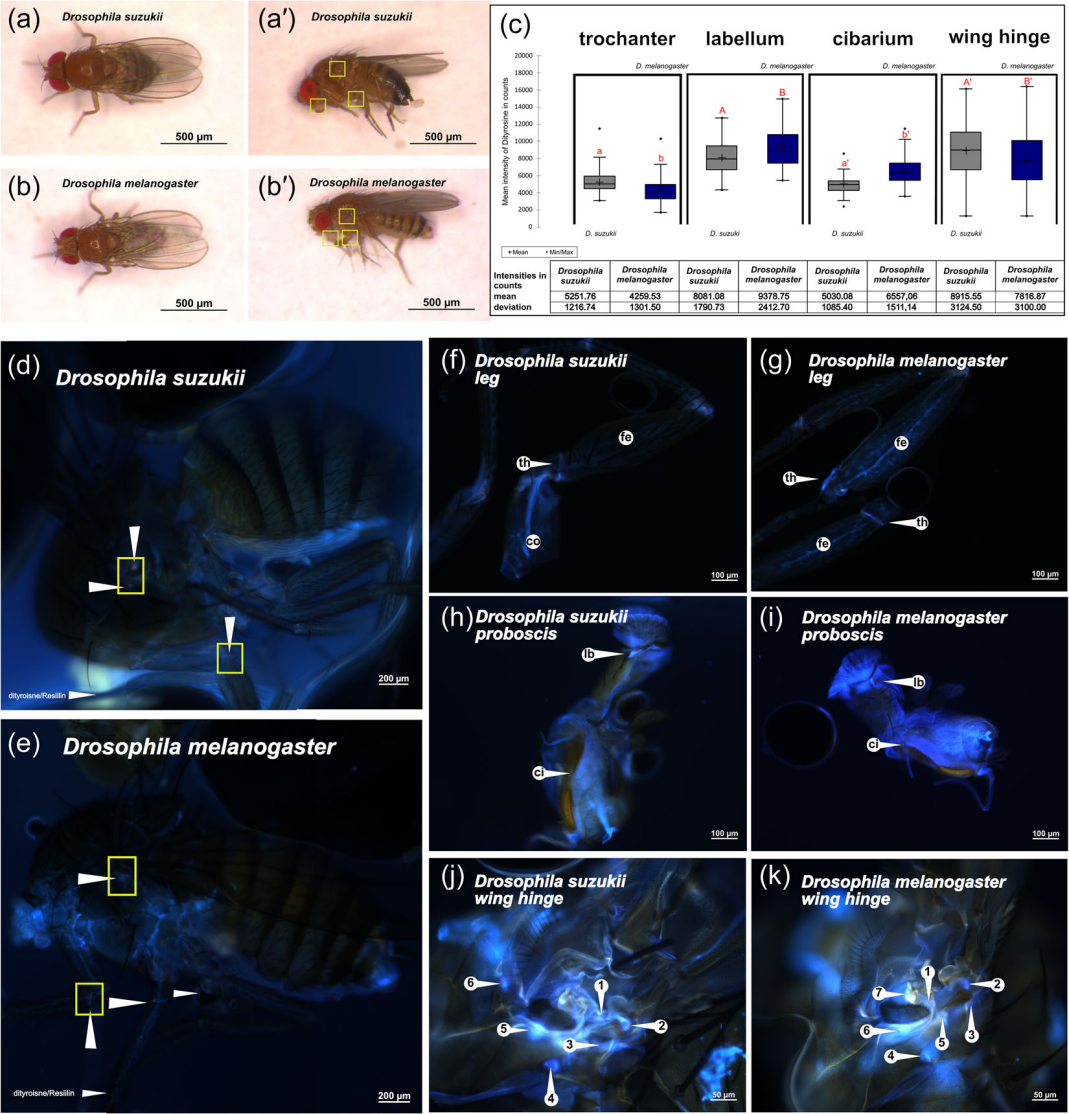
Dityrosine (DT) distribution in *Drosophila melanogaster* and *Drosophila suzukii*. Dorsal and lateral view on wild‐type *D. suzukii* (female, [a] dorsal, [a'] lateral) and *D. melanogaster* (female, [b] dorsal, [b'] lateral). Wings are kept at a dorsal position. Yellow frames refer to those positions shown in d–l. (c) DT intensities in the trochanter, the labellum, the cibarium and the wing hinge are significantly different between the two species. Data are represented as boxplots. Different letters indicate significant differences (one‐way analysis of varience followed by Tukey HSD post hoc procedure, see Supporting Information: File S4). In some regions, the DT signal seems to be stronger in *D. melanogaster* (proboscis), while in others it is stronger in *D. suzukii* (trochanter, wing hinge); *n Dm* = 84 individuals, *n Ds* = 69 individuals. Examples are shown in d–l. The whole bodies are presented in d and e. DT signals identified in the wing hinge (f), the proboscis (g) and the leg (h) of *D. suzukii* had also been identified in *D. melanogaster* (i, k, and l, respectively) by Lerch et al. (2020). Signal distribution in the wing hinge with 6–7 dots per individual was similar in both species (f and i). In general, the wing hinge regions seems to be well conserved between *D. suzukii* and *D. melanogaster*. ci, cibarium; cx, coxa; fe, femur; tr, trochanter; la, labellum.

Before studying the role of Ppro‐Resilin in the function of the Resilin matrices in *D. suzukii*, we determined the presence of Resilin in the adult fly. We compared the distribution of Resilin matrices in *D. suzukii* and *D. melanogaster*. *D. suzukii* flies are bigger than *D. melanogaster* flies (Table 1). The DT signals at the wing hinge and the trochanter are stronger in *D. suzukii* than in *D. melanogaster* (Figure 1). By contrast, the DT signals in the proboscis (labellum and cibarium) are stronger in *D. melanogaster* than in *D. suzukii*. Previously, we had already observed that the intensity of the DT signal in the wing blade of the bigger *D. hydei* is higher than in *D. melanogaster* (Lerch et al., 2020). Together, this suggests that there may be a positive correlation between DT signal intensity in some regions and body size in *Drosophila* species. In some other DT regions like the proboscis, however, this correlation does not occur.

**Table 1:** Body and wing sizes in *Drosophila suzukii* and *Drosophila melanogaster*

	*D. suzukii* female	*D. suzukii* male	*D. melanogaster* female	*D. melanogaster* male
Wing	2458.76	2184.82	2130.86	1849.23
	SD 151.14	SD 105.34	SD 29.49	SD 49.67
Body	3404.95	2828.41	2875,2	2585.14
	SD 136.76	SD 102.51	SD 93.57	SD 138.6

*Note*: The wing and body size (in µm) differences are significant between the sexes of the species according to a Student's *t*‐test: *p*female wing = 5.1408E‐06, *p*male wing =  275119E‐08, *p*female body = 169297E‐08, *p*male body = 000037385.

To attempt to clarify this issue, we extended our comparative approach to a third *Drosophila* species, *Drosophila hydei, D. hydei*, and *D. suzukii* flies have a similar body size, thus, *D. hydei* are bigger than *D. melanogaster* flies (Supporting Information: Data S4). The DT signal intensities in the trochanter and the wing hinge are not significantly different between *D. suzukii* and *D. hydei* (Supporting Information: Figure S1 and Data S4). These intensities are, however, stronger in *D. hydei* than in *D. melanogaster*. In the cibarium, the DT signal intensities significantly differ between all three species, with the strongest signal present in the smallest species *D. melanogaster*. In the labellum, we found that signal intensities do not differ between *D. melanogaster* and *D. hydei*. In these species, this signal is, however, stronger than in *D. suzukii*. In summary, we observe a trend of a stronger DT signal in body parts needed for locomotion in bigger *Drosophila* species; in the proboscis, especially the cibarium, however, the situation is rather complicated not allowing to formulate a clear working hypothesis. Apparently, besides body size, other factors seem to be decisive for DT intensities in the proboscis.

To substantiate these effects, we conceived the subtractive body size‐to‐DT intensity coefficient C^δs:dt^ (see Section 2). Most of determined C^δs:dt^ values between *D. melanogaster*, *D. suzukii*, and *D. hydei* approximated 0 suggesting a proportional body size‐to‐DT intensity correlation (Table 2 and Supporting Information: Data S4). The exception is the C^δs:dt^ for the cibarium persistently deviating from 0. Simply, the DT signal intensity in the cibarium does not seem to correlate with the body size. Thus, obviously, it depends on other factors such as the proboscis or cibarium size or the physical properties of the food. More detailed analyses of these parameters and the DT intensity in the proboscis are needed to address this problem.

**Table 2:** Body size‐to‐dityrosine (DT) intensity coefficients (C^δs:dt^) in *Drosophila melanogaster*, *Drosophila suzukii*, and *Drosophila hydei*.

	*D. suzukii* versus	*D. melanogaster*	*D. suzukii* versus
	*D. melanogaster*	versus *D. hydei*	*D. hydei*
Wing hinge	−0.00018	0.00570	0.00552
Trochanter	−0.04840	−0.00229	−0.07120
Labellum	0.09417	−0.06054	0.03363
Cibarium	0.20266	−0.64653	−0.44390

*Note*: The body size‐to‐DT intensity coefficients Cds:dt were determined according to the equation presented in Section 2. A value of 0 suggest a simple body size‐to‐DT intensity correlation. Cδs:dt values deviating from 0 suggest that other factors than body size contribute to the DT intensity.

In the literature, we found only one article reporting on this question. The correlation between body mass and Resilin volume is inversed in the thorax of planthoppers and froghoppers (Siwanowicz & Burrows, 2017). Overall, there seems to be no clear trend in the relationship of DT signal intensity and body size or mass in some insects. Additional intensive work in different insect species is needed to clarify this aspect of resilin.

### 3.2 Generation of Pro‐Resilin mutations in *D. suzukii*

**Figure 2:**
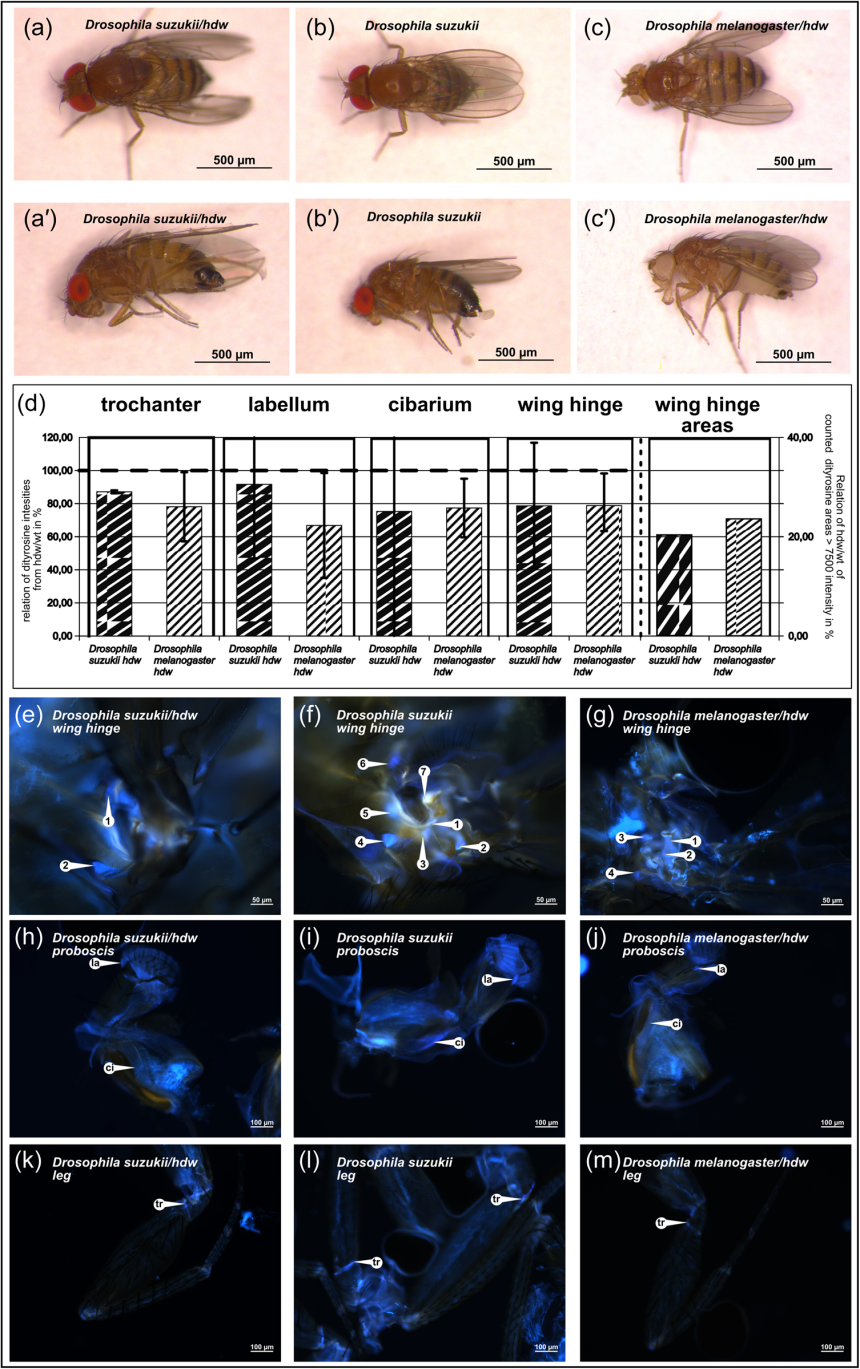
Flies homozygous mutant for *proresilin* show the *held‐down‐wing*‐phenotype in *Drosophila suzukii*. The *held‐down‐wing* phenotype (*hdw*) of *D. suzukii proresilin* mutant flies (female, [a] dorsal view, [a'] lateral view), as opposed to the wild‐type fly (b and b') resembles the *hdw*‐phenotype of *Drosophila melanogaster proresilin* mutant flies (female, [c] dorsal view, [c'] lateral view). (d) Dityrosine (DT) intensities in different body regions are reduced in *hdw* flies compared to wild‐type flies (set to 100%, the original values are shown in Supporting Information: Figure 1D) in both *D. suzukii* and *D. melanogaster*. In the trochanter, the labellum, the cibarium and the wing hinge, the DT signal intensities are reduced in the range of 8.5%–25% and 16%–33% in *D. suzukii^hdw^* and *D. melanogaster^hdw^* flies, respectively, compared to their wild‐type counterparts (n Ds*hdw*/Ds = 16/69 individuals; n Dm*hdw*/Dm = 82/84 individuals). In addition to intensity loss, in the wing hinge, we also observed a reduction of DT signal dot number in *hdw* flies—*D. suzukii* 6.3 versus *hdw* 0.9 (14.87%) and *D. melanogaster* 5.3 versus *hdw* 1.3 (24.03%). This reduction value in *D. melanogaster* is different compared to the published one of around 13% (Lerch et al., 2020). This is mainly due to the microscopy technique applied: while we used fluorescence microscopy in this study, we had obtained our data by confocal microscopy in the published work. (e–m) Examples of DT regions in *hdw* female flies. The potential *proresilin* knock‐outs still display regions with considerable DT signals. Indeed, the signal intensity differences are not per se detectable by the naked eye. However, in the wing hinge of *hdw* flies (*D. suzukii* [e]; *D. melanogaster* [g]), the number of the dots is reduced compared to the wild‐type (*D. suzukii* [f]; *D. melanogaster* Figure 1I). The proboscis signal, especially the labellum, is visible in all flies (h–j). The trochanter (*D. suzukii* [k]; *D. melanogaster* [m]) is more often a dim line compared to the situation in the respective wild‐type (*D. suzukii* [l]; *D. melanogaster* Figure 1l). Fluorescence microscopy was done on a Zeiss Axiophot equipped with a Axiocam mono camera and the respective software. ci, cibarium; cx, coxa; fe, femur; tr, trochanter; la, labellum.

**Figure 3:**
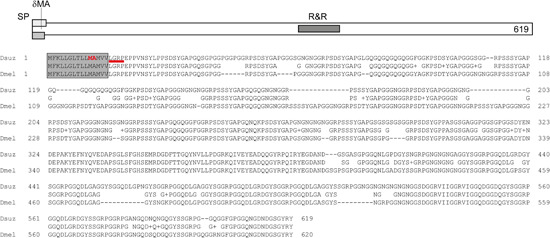
*Drosophila suzukii* Pro‐Resilin. The *D. suzukii* Pro‐Resilin protein has 619 residues with an N‐terminal signal peptide (SP) and a middle R&R‐type chitin‐binding domain (R&R). The mutation introduced by the CRISPR/Cas9 method causes a deletion of two amino acids (M_11_ and A_12_) in the signal peptide leading to a shift of the SP cleavage site to the position between P_19_ and E_20_ as predicted by the SignalP 6.0 software. The cleavage site efficiency is reduced by almost 20% c. According to the software, proresilin SPs from *D. suzukii* and *Drosophila melanogaster* are highly conserved.

To study the role of Resilin in *D. suzukii*, we applied the Crisp/Cas9 technique to introduce mutations in the *proresilin* gene. Genetic analyses of the *proresilin* locus in *D. melanogaster* had revealed that loss‐of‐function mutations are viable causing a wing posture that is, *hdw* phenotype (Lerch et al., 2020). We expected therefore to observe the same *proresilin*‐less phenotype in *D. suzukii*. In our population of flies derived from embryos subjected to Crisp/Cas9 treatment, we found flies displaying the *hdw* phenotype (Figure 2 and Supporting Information: Figure S2). These *D. suzukii* flies resemble the respective *D. melanogaster* flies. We sequenced the *proresilin* locus of *D. suzukii hdw* flies and identified a deletion mutation within the gDNA recognition sequence (Figure 3). This deletion removes two codons coding for an M_11_ and an A_12_ at the positions 31–36 of the coding sequence. These two amino acids are part of the predicted signal peptide sequence from residues 1–15 (cleavage site probability of 0.982695) that is needed for the secretion of Pro‐Resilin to the extracellular space. According to the SignalP 6.0 software, the deletion of M_11_A_12_ would shift the signal peptide cleavage site to the position between residues 17 and 18 concomitantly reducing the probability of signal peptide cleavage (0.810029). The new cleavage site, if functional, would cause the deletion of four N‐terminal amino acids of the secreted protein. We assume that disruption of the signal peptide prevents the efficient secretion of Pro‐Resilin into the differentiating cuticle, thereby causing the observed phenotype. In addition, N‐terminal truncation of the protein might destabilise it and/or reduce its activity. Together, despite the deletion, low amounts of aberrant Pro‐Resilin may be secreted correctly and display residual function, thereby explaining the relatively mild nonlethal phenotype. Alternatively, redundant DT‐forming proteins may act in parallel to Pro‐Resilin thereby alleviating the defect. Indeed, the genomes of many insect species harbour more than one gene coding for Pro‐Resilin‐like proteins (Andersen, 2010).

### 3.3 DT signals are reduced in proresilin mutant *D. suzukii*

To evaluate the effects of the *proresilin^CC^* mutation on the cuticle quality, we compared the DT signal intensities in control and *proresilin^CC^* flies. In wild‐type flies, DT was mainly detected in the proboscis, the wing hinge, the spermatheca, the base of bristles and at different positions in the leg, for example, in the trochanter (Figure 1 and S3). In *proresilin^CC^* flies, the DT signal intensities were significantly reduced by 8.5% (labellum), 13% (trochanter), 16% (wing hinge), and 25% (cibarium) (Figure 2 and Supporting Information: Figure S2). The DT intensity differences between *proresilin^CC^* and wild‐type flies are in general below the intensity differences in respective *D. melanogaster* flies (22% trochanter and wing hinge, 25% cibarium, and 33% labellum) (Figure 2D and Supporting Information: Figure S2D). In other words, the *proresilin^CC^* mutation has a less severe impact on DT intensity in *D. suzukii* compared to *D. melanogaster*. However, in the wing hinge, reduction of the number of DT dots is slightly higher in *D. suzukii* than in *D. melanogaster*. These differences in DT intensity and distribution may be due to the different types of mutations. In *D. melanogaster* the *proresilin* mutations generate loss‐of‐function proteins (Lerch et al., 2020), while in *D. suzukii* a truncated protein might be produced and probably to a lower extent secreted. Nonelimination of the DT signal may, as in *D. melanogaster*, alternatively be explained by the presence of other DT‐forming proteins in the resilin matrix including Cpr56F (Ardell & Andersen, 2001; Lerch et al., 2020). Indeed, *cpr56F* is present in the *D. suzukii* genome (spottedwingfly.org: DS10_00002334) and conserved in other *Drosophila* species (orthology ID 2370). As Cpr56F is, probably like in *D. melanogaster*, not expressed in the wing hinge, at least a third DT‐forming protein may be postulated to be incorporated into Resilin matrices in at least some body parts in *Drosophilae*. Overall, based on our data, we conclude that the Resilin matrix composition and probably function are largely conserved between *D. melanogaster* and *D. suzukii*. Despite this, we detected minor differences in DT signal intensity and distribution (e.g., in the wing hinge) suggesting that resilin matrices may be optimized in related species during evolution to accommodate slightly different lifestyles. The biomechanics consequences of these differences, once investigated, may serve to advance in biomimetics approaches.

## AUTHOR CONTRIBUTIONS

Steven Lerch performed most histological experiments, analysed the data and contributed to the writing of the first draft of the manuscript. Yang Yang assisted Steven Lerch in microscopy in some experiments. Justin Flaven‐Pouchon performed the statistical analyses of the data. Nicole Gehring generated the CRISPR/Cas9 flies. Bernard Moussian conceived and planned all experiments. He analysed the data and wrote the manuscript.

## CONFLICTS OF INTEREST

The authors declare no conflicts of interest.

## Supporting information

Supporting information.

Supporting information.

Supporting information.

Supporting information.

Supporting information.
