# Supplementary figures and images for: Resilin is needed for wing posture in Drosophila suzukii
Source: Arch Insect Biochem Physiol. 2022 May 23;111(1):e21913. doi: 10.1002/arch.21913 (PMC9539844; doi:10.1002/arch.21913)

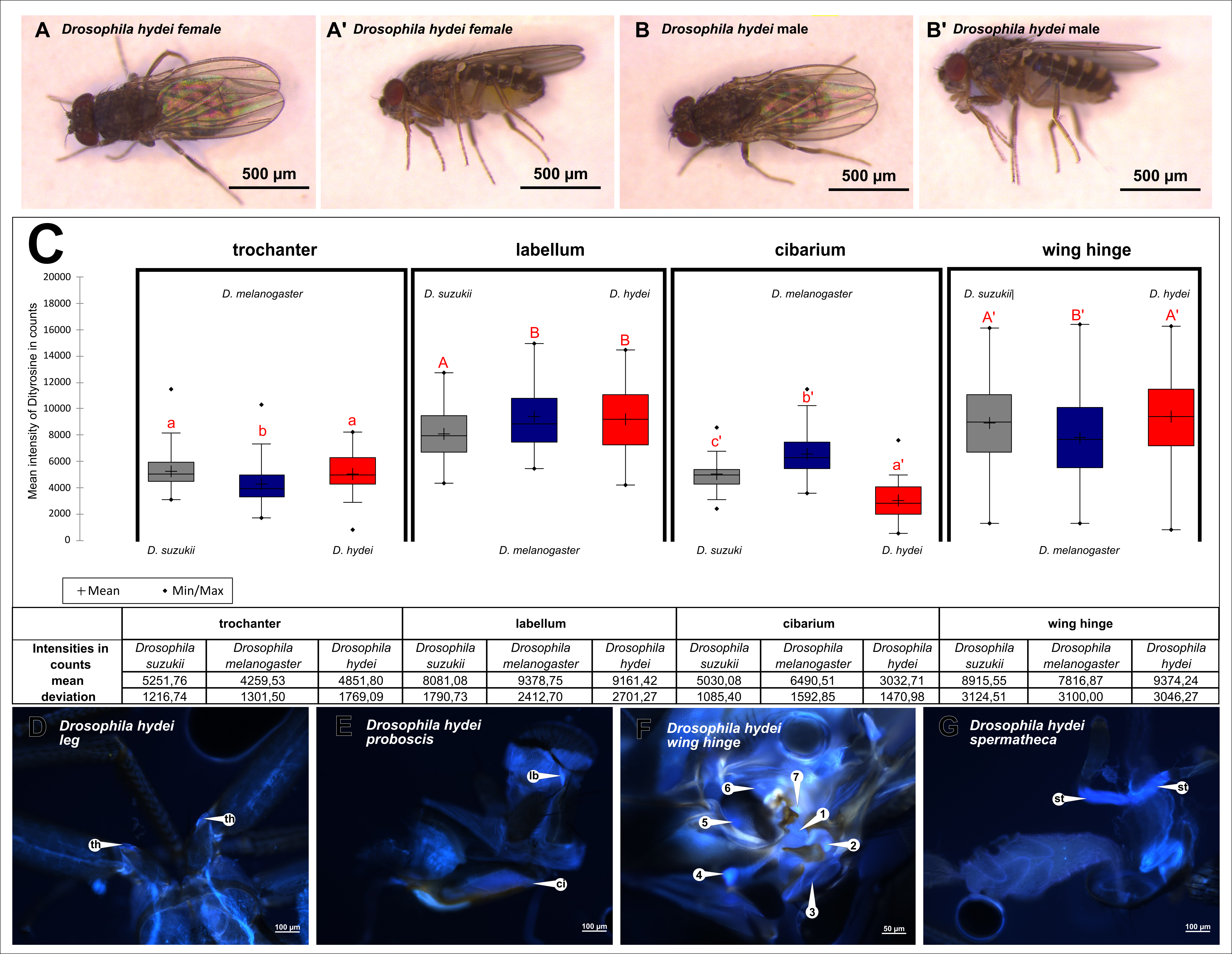

Supplement: Supplementary file 1 — Supporting information. [file ARCH-111-e21913-s002.png]

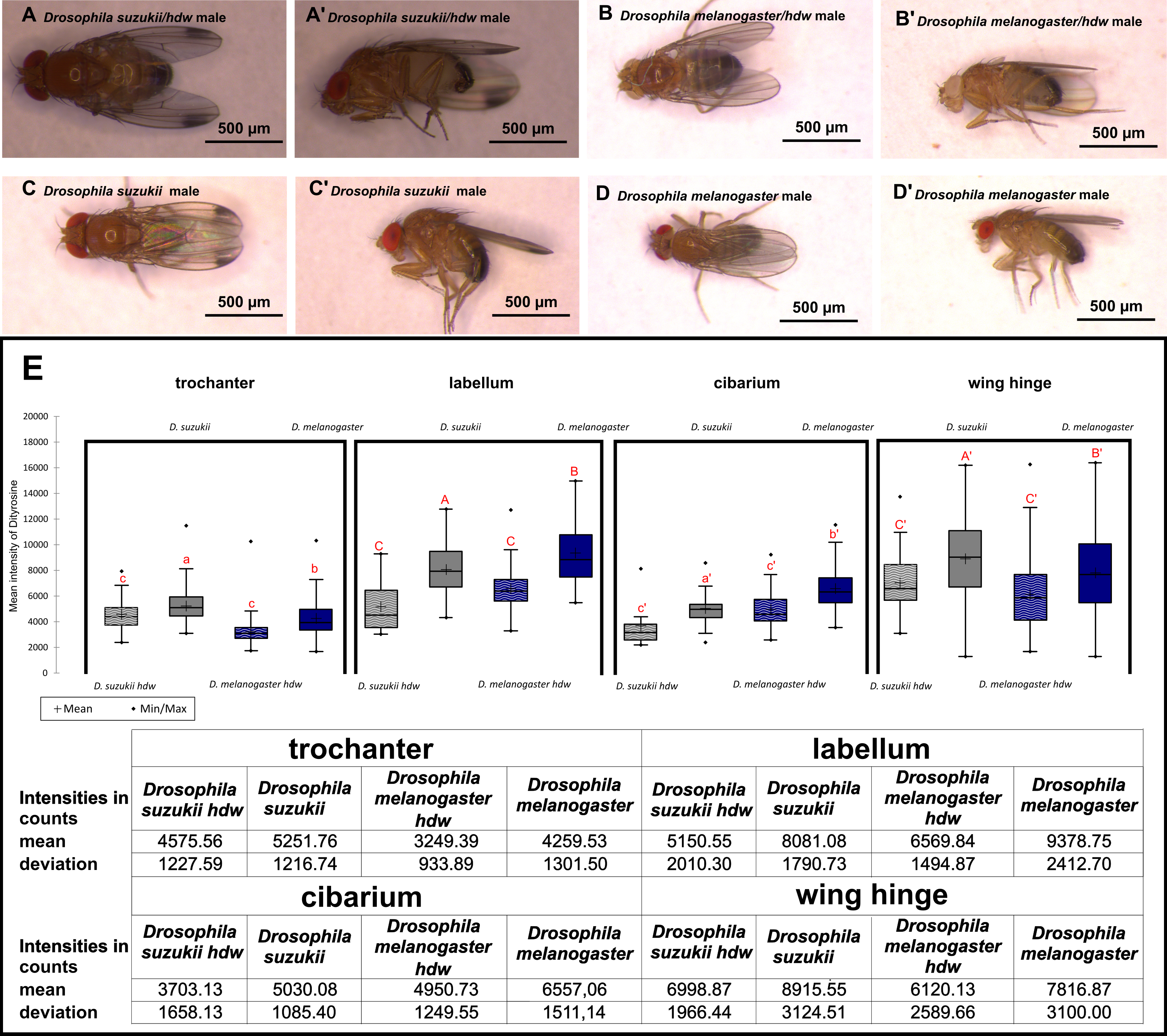

Supplement: Supplementary file 2 — Supporting information. [file ARCH-111-e21913-s004.png]

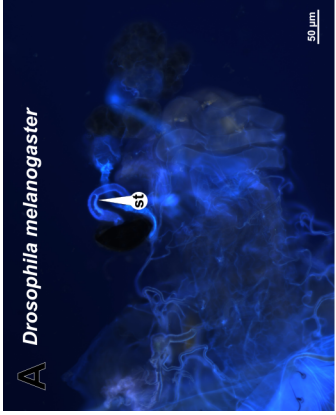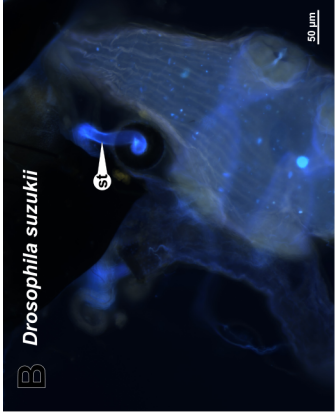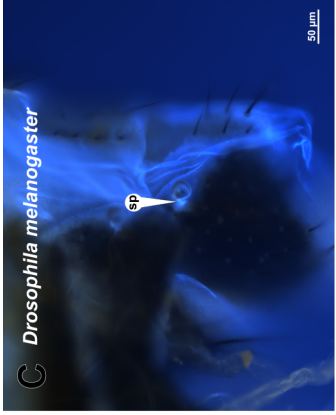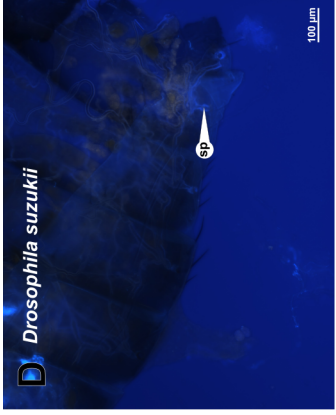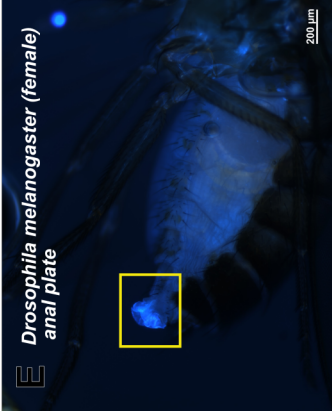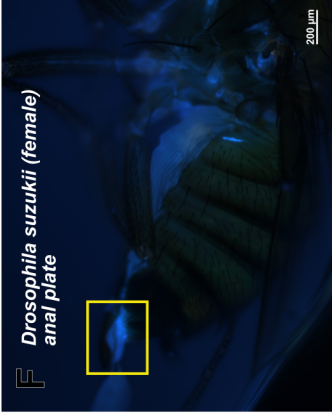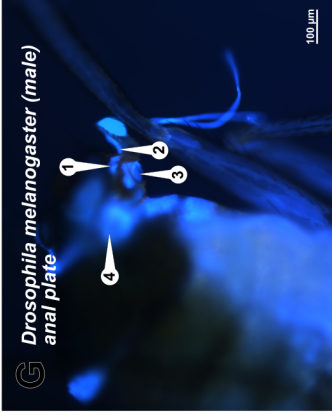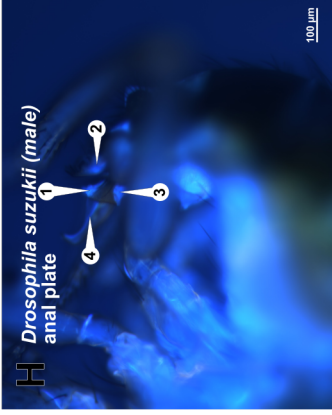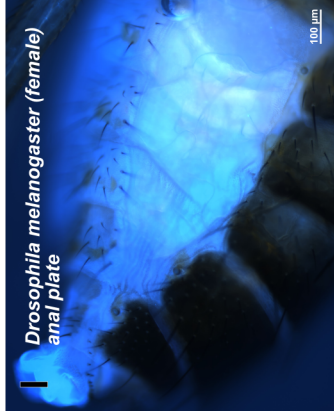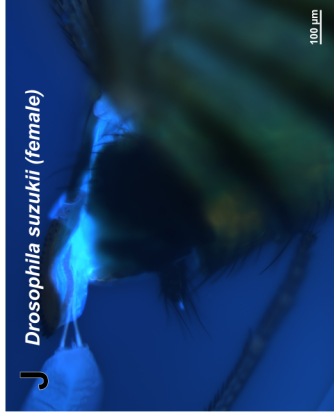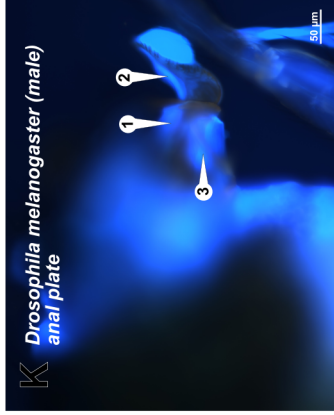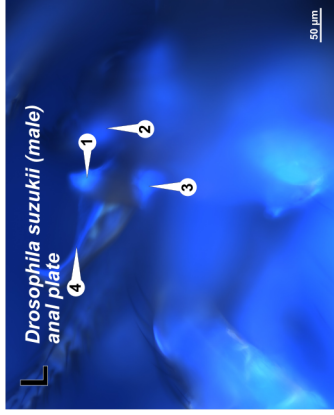

Supplement: Supplementary file 3 — Supporting information. [file ARCH-111-e21913-s003.pdf]
