# Supplementary material for: Resilin is needed for wing posture in Drosophila suzukii
Source: Arch Insect Biochem Physiol. 2022 May 23;111(1):e21913. doi: 10.1002/arch.21913 (PMC9539844; doi:10.1002/arch.21913)
Supplement: Supplementary file 4 — Supporting information. [file ARCH-111-e21913-s001.pdf]

**student t.test**	**wing hinge**				
**species / species**	*D. melanogaster / D. suzukii*	*D. melanogaster / D. hydei*	*D. hydei / D. suzukii*	*D. melanogaster wt/hdw*	*D. suzukii wt/hdw*
**p value=**	1,44514E-11	5,34712E-85	0,059777181	4,57006E-21	4,45963E-06
**significant**					
**student t.test**	**trochanter**				
**species / species**	*D. melanogaster / D. suzukii*	*D. melanogaster / D. hydei*	*D. hydei / D. suzukii*	*D. melanogaster wt/hdw*	*D. suzukii wt/hdw*
**p value=**	3,06167E-13	0,007536933	0,074512822	1,08148E-17	0,002694876
**significant**					
**student t.test**	**labellum**				
**species / species**	*D. melanogaster / D. suzukii*	*D. melanogaster / D. hydei*	*D. hydei / D. suzukii*	*D. melanogaster wt/hdw*	*D. suzukii wt/hdw*
**p value=**	0,000165436	0,549961896	0,018758461	5,22191E-22	1,3688E-07
**significant**					
**student t.test**	**cibarium**				
**species / species**	*D. melanogaster / D. suzukii*	*D. melanogaster / D. hydei*	*D. hydei / D. suzukii*	*D. melanogaster wt/hdw*	*D. suzukii wt/hdw*
**p value=**	1,1315E-14	3,26771E-13	1,02385E-11	3,57683E-18	0,00010881
**student t.test**	**body size**				
**species / species**		*D. melanogaster / D. suzukii*	*D. melanogaster / D. hydei*	*D. hydei / D. suzukii*	
**p value=**		2,42E-05	6,84E-13	0,120400131	
**student t.test**	**wing blade size**				
**species / species**		*D. melanogaster / D. suzukii*	*D. melanogaster / D. hydei*	*D. hydei / D. suzukii*	
**p value=**		2,21877E-07	5,11E-25	1,41E-09	
