# Supplementary material for: Resilin is needed for wing posture in Drosophila suzukii
Source: Arch Insect Biochem Physiol. 2022 May 23;111(1):e21913. doi: 10.1002/arch.21913 (PMC9539844; doi:10.1002/arch.21913)
Supplement: Supplementary file 5 — Supporting information. [file ARCH-111-e21913-s005.docx]

**Supplementary data**

**S1** Detection of DT intensities in *Drosophila hydei* wild-type flies.

Dorsal and lateral views of *Drosophila hydei* females and males (A-B).

For comparison, the intensity values of the DT signal in the trochanter, the labellum, the cibarium and the wing hinge in *D. hydei* are joined with the respective values in *D. melanogaster* and *D. suzukii* shown in Figure 1C (C). In *D. hydei* flies, DT was detected in the trochanter (D), the proboscis (E), the wing hinge (F) and the spermatheca (G).

tr… trochanter, ci… cibarium, la… labellum, st... spermatheca.

**S2.** DT intensities in male wild-type and *hdw* *D. melanogaster* and *D. suzukii* flies.

The wing posture phenotype in dorsal and lateral views of *hdw* flies (*hdw* A, B; control C, D) is identical to the phenotype in females shown in figure 2. (E) The graphs show the actual DT signal intensities quantified by fluorescence microscopy (with the values listed below) for the trochanter, the labellum, the cibarium and the wing hinge for females and males. Different letters indicate significant differences (One-way ANOVA followed by Tukey HSD post-hoc procedure).

**S3.** Additional areas of DT in the abdomen of *D. melanogaster* and *D. suzukii*.

A and B: spermatheca (st). C and D: spiracles (sp). E-H: anal plates of both sexes at low magnification. I and J: In the female, the DT signal is diffuse from outside at high magnification. K and L: by contrast, in the male, distinct areas of DT are visible at the same magnification. Fluorescence microscopy was done on a Zeiss Axiophot equipped with a Axiocam mono camera and the respective software.

**S4** Statistic data of all experiments in this work including p-values.
